# Technology Activities and Cognitive Trajectories Among Community-Dwelling Older Adults From the NHATS 2015-2023

**DOI:** 10.1093/geroni/igaf122.2568

**Published:** 2025-12-31

**Authors:** Erh-Chi Hsu, Eric Jutkowitz

**Affiliations:** Johns Hopkins University, Baltimore, Maryland, United States; University of Minnesota, Minneapolis, Minnesota, United States

## Abstract

This study examines the associations between the onsets and cessations of technology use and cognitive trajectories among community-dwelling older adults without dementia. While using digital technology is positively associated with cognitive function, the impacts of specific activities on distinct cognitive domains are underexplored. Data were collected from 5,566 older adults involved in the National Health and Aging Trends Study (NHATS) from 2015 to 2023. Assessed technology activities included online shopping, banking, medication refills, social media use, and checking health conditions online. Cognitive domains measured were episodic memory, executive function, and orientation. Linear mixed-effects models analyzed associations between technology activity transitions and cognitive outcomes, adjusting for demographic, socioeconomic, and health-related factors. Onsets of online shopping (β = 0.044**), medication refills (β = 0.070***), and social media (β = 0.065**) were associated with improved episodic memory. Conversely, cessations of online banking (β=-0.083**) and social media (β=-0.064**) related to decreased episodic memory. Initiating instrumental, social, and health-related technology activities mitigated cognitive decline in orientation. Online shopping onset mitigated episodic memory decline (β = 0.013*) while cessation worsened it (β=-0.022*); a similar trend occurred with social media cessation (β=-0.018*). More device ownership consistently linked to better cognitive functioning across activities. Higher educational attainment also correlated with superior cognitive domain performance, indicating potential disparities. Results suggest engagement in technology activities supports cognitive health in older adults. Future interventions should promote initiation and sustainment of these activities to mitigate cognitive decline in aging populations.

